# Identification of a Subtype of Poorly Differentiated Invasive Ductal Carcinoma of the Breast Based on Vimentin and E-cadherin Expression

**DOI:** 10.1055/s-0038-1673700

**Published:** 2018-10-25

**Authors:** Leonardo Fleury Orlandini, Francisco José Cândido dos Reis, Willian Abraham da Silveira, Marcelo Guimarães Tiezzi, Jurandyr Moreira de Andrade, Alfredo Ribeiro-Silva, Ryan Deaton, Maarten Bosland, Daniel Guimarães Tiezzi

**Affiliations:** 1 Breast Disease Division, Department of Gynecology and Obstetrics, School of Medicine, Hospital das Clínicas da Universidade de São Paulo, Ribeirão Preto, SP, Brazil; 2 University of Illinois at Chicago, Chicago, IL, United States

**Keywords:** breast cancer, epithelial-mesenchymal transition, vimentin, e-cadherin, prognostic factors, câncer de mama, transição epitelial-mesenquimal, vimentina, e-caderina, fatores prognósticos

## Abstract

**Objective** The use of molecular markers can identify a subgroup of tumors with distinct recurrence patterns. The present study aimed to characterize the immunohistochemical expression of vimentin (VIM), of E-cadherin (CDH1), and of cytokeratin 5 (CK5) in patients with invasive ductal carcinomas (IDCs).

**Methods** We have constructed a tissue microarray (TMA) from 87 patients with IDC of the breast. Immunohistochemistry (IHC) was performed to study the expression of estrogen and progesterone receptors (ER and PgR), human epidermal growth factor receptor 2 (HER2), VIM, CDH1, CK5, and Ki67. The tumors were classified as luminal A and B (*n* = 39), HER2 enriched (*n* = 25), and triple-negative (TNBC) (*n* = 23), based on the IHC expression.

**Results** We have observed that luminal A and B tumors lack the VIM^+^/CDH1^-/low^ phenotype. This phenotype was observed in 16.5% of the HER2+ tumors and in 60% of the TNBC tumors (*p* = 0.0001). Out of a total of 20 TNBC tumors, the CK5 (basal-like marker) was positive in 11 of them. The VIM^+^/CDH1^-/low^ phenotype was observed in 5 CK5+ TNBC tumors (45%) and in 7 out of 9 CK5- TNBC tumors (78%) (*p* = 0.02). The median Ki67 index in the VIM^+^/CDH1^-/low^ tumors was 13.6 (range: 17.8–45.4) compared with 9.8 (range: 4.1–38.1) in other tumors (*p* = 0.0007). The presence of lymph node metastasis was less frequent in patients with VIM^+^/CDH1^-/low^ tumors (23% versus 61%; *X^2^* test; *p* = 0.01).

**Conclusion** Our findings suggest that the expression of VIM and CDH1 can identify a subset of IDCs of the breast with a mesenchymal phenotype associated with poor prognosis, high-grade lesion, and high mitotic index.

## Abstract (pt)

**Resumo:**

**Objetivo** O uso de marcadores moleculares pode identificar subtipos tumorais com diferentes taxas de recidiva. O objetivo do presente estudo é caracterizar a expressão imunohistoquímica da vimentina (VIM), da E-caderina (CDH1) e de CK5 em pacientes com carcinoma ductal invasivo (CDI) da mama.

**Métodos** Utilizamos uma matriz de amostras teciduais (TMA, na sigla em inglês) de 87 pacientes com CDI da mama. Para avaliar a expressão dos receptores de estrogênio (RE) e receptores de progesterona (RP), HER2, VIM, CDH1, CK5 e Ki67, utilizamos imunohistoquímica. Os tumores foram classificados como luminal A e B (*n* = 39), HER2+ (*n* = 25) e triplo negativo (TNBC) (*n* = 23).

**Resultados** Foi observado que tumores luminais A e B não expressaram o fenótipo VIM^+^/CDH1^-/low^. Este fenótipo foi observado em 16,5% dos tumores HER2+ e em 60% dos tumores TNBC (*p* = 0,0001). Dos 20 tumores TNBC, a CK5 (marcador de tumor basalóide) foi super expressa em 11 amostras. O fenótipo VIM^+^/CDH1^-/low^ foi observado em 5 tumores CK5+ TNBC (45%) e em 7 dos 9 tumores CK5- TNBC (78%) (*p* = 0,02). A expressão média de Ki67 nos tumores VIM^+^/CDH1^-/low^ foi 13.6 (amplitude de 17,8 a 45,4) comparado com 9,8 (amplitude de 4,1 a 38,1) nos outros tumores (*p* = 0,0007). A presença de metástase linfonodal foi menor em tumores com fenótipo VIM^+^/CDH1^-/low^ (23% contra 61%; teste *X^2^*; *p* = 0,01).

**Conclusão** Nossos achados sugerem que a expressão de VIM e CDH1 pode identificar um subtipo de CDI da mama com fenótipo mesenquimal associado a pior prognóstico, lesões de alto grau e alto índice mitótico.

## Introduction

Based on its genomic profile, breast cancer can be classified in six subtypes (luminal A, luminal B, human epidermal growth factor receptor 2 (HER2)-enriched, basal-like, claudin-low, and normal-like).^1^ ^2^ This classification stratifies patients with adverse prognostic characteristics, and the basal-like and claudin-low subtypes confer the shortest overall survival.^3^ ^4^ ^5^ ^6^ Immunohistochemical phenotyping based on estrogen and progesterone receptors (ER and PgR), HER2, and Ki67 expression has been proposed as an alternative to classify breast cancer into different subtypes. According to this immunophenotyping approach, breast cancer can be divided into luminal A (HR + , HER2-, low Ki67 index), luminal B (HR+ with HER2+ or HER2- plus high Ki67 index), HER2-enriched (HR-, HER2 + ) and triple negative (TNBC) (HR- and HER2-).^7^ ^8^ ^9^ ^10^

Triple negative tumors tend to have a worse prognosis than luminal A, luminal B, and HER2-enriched tumors, and no specific targeted TNBC therapy has been developed yet.^11^ ^12^ ^13^ ^14^ Although TNBC has been characterized as a pathological entity, there is significant biological heterogeneity within these tumors.^13^ ^15^ The basal-like phenotype is reported as the main TNBC subtype.^8^ ^16^ ^17^ However, all the other molecular subtypes can occur.^16^ ^17^ The use of immunohistochemistry (IHC) to identify different subgroups of TNBC tumors has been proposed. Cytokeratin 5 (CK5) and epidermal growth factor receptor (EGFR) expressions have been reported as markers of basal-like carcinomas.^7^ ^8^ ^14^ ^15^ ^18^ In fact, CK5 expression has been reported to be the most accurate basal-like marker.^7^ ^19^ ^20^ ^21^ ^22^ Breast cancers that have arisen from basal cells are supposed to have a mesenchymal phenotype.^17^ ^23^ ^24^ The claudin-low group lacks cell-cell junction proteins, and has features resembling the epithelial-mesenchymal transition (EMT) activation.^2^ ^13^ ^17^ ^25^ Additionally, some studies have suggested that there is a subgroup with a quintuple-negative profile (HR-, HER2-, CK5-, and EGFR-) that exhibits a worse prognosis when compared with CK5+ or EGFR+ TNBC.^18^ ^26^ Thus, the use of EMT markers can identify an undifferentiated subgroup with a possibly highly aggressive clinical behavior.^4^ ^17^ ^27^

Most investigated and reported EMT markers include the E-cadherin (CDH1), a cell surface protein responsible for the adhesion of epithelial cells. Downregulation in CDH1 is highly associated with EMT, and partial or total loss of CDH1 expression is associated with more aggressive behavior and poor prognosis.^28^ ^29^ ^30^ Often coupled with low CDH1expression, vimentin (VIM), a type III intermediate filament protein that is expressed in mesenchymal cells, has been used to investigate EMT activation in epithelial tissues. The VIM expression in epithelial cells is associated with a migratory phenotype and consequent invasiveness and metastasis.^29^ ^31^ ^32^ ^33^ The aim of the present study was to determine the VIM and CDH1 expression in invasive ductal carcinomas (IDCs) of the breast, and to analyze their association with CK5 expression and clinical and pathological features.

## Methods

### Sample Selection

We have selected all patients (*n* = 175) with IDC of the breast subjected to surgery at Hospital das Clínicas of the Faculdade de Medicina de Ribeirão Preto between January 2005 and December 2007. The selection was based on the histological and immunohistochemical diagnosis in the files of the patients. The staging system was based on the American Joint Committee on Cancer (7^th^ edition) classification. We have used pathological staging for patients subjected to primary surgery, and clinical staging in cases of neoadjuvant treatment. According to the IHC reports, 27 IDCs were TNBC, 39 were HER2+ (with a score of 3 +), 36 were HER2+ (score of 2 +), and 73 were HR+ (for estrogen or progesterone receptors) and HER2- (with a score of 0 or 1 +). We were able to retrieve 94 paraffin blocks, and 82 were suitable for tissue microarray (TMA) construction. The total sample comprised 24 TNBCs, 9 HER+ and HR-, 14 HER2+ and HR + , and 35 HR+ and HER2-. All samples were reevaluated, and the histological diagnosis and tumor grade were reported. Table 1 summarizes the characteristics of the patients.

**Table 1:** Characteristics of 82 patients with invasive ductal carcinoma of the breast

Parameter	* n *= 82
Age (mean ± SD)	54.4 ± 13.1
Menopausal status	
Pre	29
Post	53
Clinical Stage	
I	8
IIA	43
IIB	16
IIIA	8
IIIB	6
IV	1
Histological grade	
1	9
2	49
3	24
Neoadjuvant treatment	
Chemotherapy	28
Hormone therapy	3
No	51
Estrogen receptor	
Positive	49
Negative	33
Progesterone receptor	
Positive	44
Negative	38
HER2	
Positive	23
Negative	59

Abbreviation: HER2, human epidermal growth factor receptor 2.

### Tissue Microarray

Core biopsies (diameter = 1mm) were punched from 2 representative areas of each tumor of each of the 87 donor paraffin blocks and arrayed into a new recipient paraffin block using a manual tissue arrayer (Beecher Instruments, Sun Prairie, WI, USA). Three 1 μm-thick sections were cut from a tissue microarray (TMA) paraffin block using a paraffin tape-transfer system (Instrumedics Inc., Saint Louis, MO, USA). The slides were dipped in paraffin to prevent oxidation. One section was stained with hematoxylin and eosin (H&E) to confirm the presence of the tumor by light microscopy.

### Immunohistochemistry

The immunohistochemical staining was performed using the Novolink Max Polymer Detection System (Leica Biosystems, Wetzlar, Germany). The sections were deparaffinized in xylene and rehydrated through a series of graded alcohols. The endogenous peroxidase activity was blocked for 30 minutes in a solution containing 0.3% of hydrogen peroxide to block non-specific immunoassaying. The sections were then placed in a 10 mM citrate buffer and submitted to heat retrieval using a vapor lock for 40 minutes. After the antigen retrieval, the specimens were allowed to cool for 30 minutes, and then incubated at 4° C overnight with a primary antibody. The dilution and source of the primary antibodies used in the present study were: anti-human ER (1:100, clone 6F11) (Leica Biosystems, Wetzlar, Germany), anti-human PgR (1:100, clone 1A6) (Leica Biosystems, Wetzlar, Germany), anti-human-Ki67 (1:200) (Leica Biosystems, Wetzlar, Germany), anti-human CK5 (HPA024467, 1:100) (Sigma-Aldrich, St. Louis, MO, USA), anti-human vimentin (1:100, clone V9) (Biocare Medical, Pacheco, CA, US), and anti-human CDH1 (1:50, clone HECD-1) (Biocare Medical, Pacheco, CA, USA). After overnight incubation with the primary antibody, the slides were incubated with a postprimary solution for 30 minutes and then incubated with the polymer for another 30 minutes (both provided by the Novolink Max Polymer Detection System). The reaction was developed with diaminobenzidine (DAB), followed by hematoxylin counterstaining. The slides were then dehydrated in an ethanol series and mounted with Permount (Fischer, Fair Lawn, NJ, USA). The DAKO Herceptest (Agilent Technologies, Santa Clara, CA, USA) was used for HER2 protein staining following the protocol of the manufacturer.

Digital analysis: In brief, the Ki67 TMA slide was scanned at 20 × magnification on an Aperio Scanscope CS (Aperio Technologies Inc., Vista, CA, USA). The Aperio Scanscope CS is a whole-slide imaging system that scans entire tissue sections and registers the image stripes into one file. The image was uploaded onto an Aperio Spectrum Plus server, and then we used the TMA Laboratory module to break the image into its logical components of sectors, rows, columns, and cores. The Nuclear V9.1 (Aperio Technologies, Vista, CA) image analysis algorithms were used for the quantification of the Ki67 staining. In general, this algorithm begins by splitting the color data in the image into a maximum of 3-color channels. In this case, we separated the channels into a blue hematoxylin channel and a brown DAB channel. The remaining channel was left at its default red color and was ignored in the analysis. For nuclear staining of the Ki67 protein, the colocalization algorithm reports the percentage of positive cells within all cancer cells.

### Interpretation of Immunohistochemical Staining

**Fig. 1:**
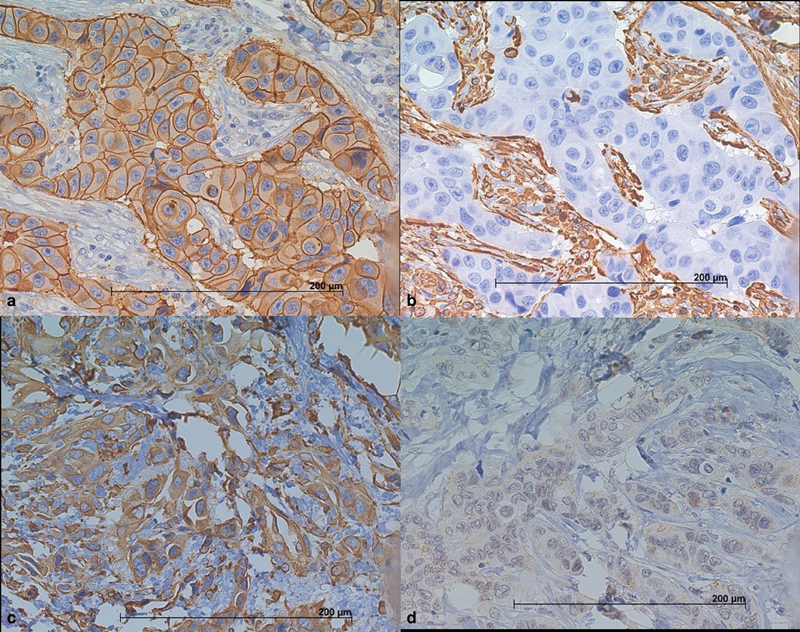
Expression of vimentin (VIM) and E-cadherin (CDH1) in invasive ductal carcinoma of the breast. The figures a and b illustrate a grade 2, luminal A invasive ductal carcinoma with a strong positive membrane staining for CDH1 (a) and negative expression of VIM (b). Note the VIM expression in stromal cells and the complete lack of staining in epithelial malignant cells. Figures c and d illustrate a grade 3, TNBC with VIM^+^/CDH1^-/low^ phenotype (vimentin positive in c and CDH1 negative in d). Note the positive cytoplasmic staining for vimentin. Expression of CDH1 is absent in cell membrane.

The cases were interpreted as positive for ER and PgR if the Allred score was ≥ 3.^34^ Marker Ki67 was considered positive if > 13% of the cancer cells in a core were stained (digital analysis). The expression of HER-2 was scored according to the degree and proportion of membrane staining as per the Herceptest protocol. The ER, PgR, and HER2 positive expression rates were 86.1%, 72.2%, and 25%, respectively. The tumors were then classified according to ER, PgR, Ki67, and HER2 protein expression as luminal A (ER+ or PgR + , and HER2-), luminal B (ER+ or PgR + , with HER2+ or Ki67 ≤ 13%), HER2+ (ER- and PgR-, with HER2 + ), and TNBC (ER-, PgR-, and HER2-). Vimentin was considered positive if > 30% of the tumor cells in a core exhibited cytoplasmic brown DAB staining. E-cadherin was denominated as CDH1^-/low^ if there was no membranous staining at all or if there were > 30% of tumor cells with no staining or discontinued membranous staining in a core. Due to missing cores on slides from the TMA block, the analysis of VIM and CDH1 expression was satisfactory in 73 samples; Ki67 and CK5 expression was satisfactory in 82 and 81 samples, respectively. The ER, PgR, and HER2 expression was analyzed in 82 samples. Fig. 1 shows positive and negative expressions for VIM and CDH1.

### Statistical Analysis

The expression of VIM and CDH1proteins and other categorical variables were compared using the standard *X^2^* test or the Fisher exact test. The difference in the Ki67 staining index was evaluated using the median test, while the difference in age between groups was assessed using the *t*-test. The disease-free and overall survival interval was calculated from the date of diagnosis, and the survival curves were derived from Kaplan-Meier estimates and compared by log-rank tests. The JMP version 10 software (SAS, Cary, NC, USA) was used for the statistical analyses. The significance was established as *p* < 0.05 (two-sided).

## Results

### Expression of CK5, VIM and CDH1 in IDCs

According to the immunophenotyping performed on TMA, 30 samples were classified as luminal A, 19 as luminal B, 9 as HER2-enriched, and 24 as TNBC. The CK5 expression was more frequent in TNBC tumors (13 out of 24 tumors, 54.2%), and it was positive in 3 luminal A tumors (10%), in 1 luminal B tumor (5.3%), and only in 1 (11%) HER2-enriched tumor (*x^2^* test; *p* = 0.0002). Vimentin was not expressed in luminal A and HER2-enriched tumors, but positive expression was observed in 71.4% of the TNBC tumors and in 5.3% of the luminal B tumors (*X^2^* test; *p* < 0.0001). The absence or reduction in CDH1 expression (CDH1^-/low^) was not observed in luminal B and in HER2 enriched tumors, but it was observed in 7.1% and 61.9% of the luminal A and TNBC tumors, respectively (*x^2^* test; *p* < 0.0001).

### VIM^+^/CDH1^-/low^ phenotype in invasive ductal carcinomas (Table 2)

We have analyzed the combined expression of VIM and CDH1 and observed that luminal A, luminal B, and HER2-enriched tumors lack the VIM^+^/CDH1^-/low^ phenotype, whereas this phenotype was observed in 61.9% of the TNBC tumors (*x^2^* test; *p* < 0.0001). The VIM^+^/CDH1^-/low^ phenotype was observed in 5 out of 15 CK5+ TNBC tumors (33%), and in 8 out of 57 CK5- TNBC tumors (14%) (Fisher exact test; *p* = 0.13). The median Ki67 index in VIM^+^/CDH1^-/low^ tumors was 13.6, compared with 9.8 in other tumors (median test; *p* = 0.0007).

**Table 2:** Clinical and pathological characteristics of 73 invasive ductal carcinomas of the breast according to the VIM^+^/CDH1^-/low^ phenotype

	VIM^+^/CDH1^-/low^	Other	*p*-*value*
Age (mean ± SD)	55.9 ± 16.1	53.5 ± 12.0	0.6
Menopausal status			
Pre	5	23	
Post	8	37	0.9
Clinical stage			
I	1	6	
II	9	45	
III/IV	3	9	0.7
Histological grade			
1	0	9	
2	4	40	
3	9	11	0.0009
Axillary status			
Positive	3	37	
Negative	10	23	0.01
Estrogen receptor			
Positive	0	43	
Negative	13	13	< 0.0001
Progesterone receptor			
Positive	0	42	
Negative	13	18	< 0.0001
HER2			
Positive	0	19	
Negative	13	41	0.01
Ki67 (median, range)	13.6 (17.8–45.4)	9.8 (4.1–38.1)	0.0007
CK5			
Positive	5	10	
Negative	8	49	0.13
Tumor subtype			
Luminal A	0	28	
Luminal B	0	19	
HER2 enriched	0	5	
TNBC	13	8	< 0.0001

Abbreviations: CDH1, E-cadherin; CK5, cytokeratin 5; HER2, human epidermal growth factor receptor 2; SD, standard deviation; TNBC, triple-negative breast cancer; VIM, vimentin.

Comparing tumors with the VIM^+^/CDH1^-/low^ phenotype with the other tumors, there was no difference in the age, menopausal status, clinical stage, and the presence of lymph node invasion in the patients (*X^2^* test; *p* = 1.0). The frequency of lymph node metastases was lower in patients with VIM^+^/CDH1^-/low^ tumors than in other tumors (Table 2).

**Fig. 2:**
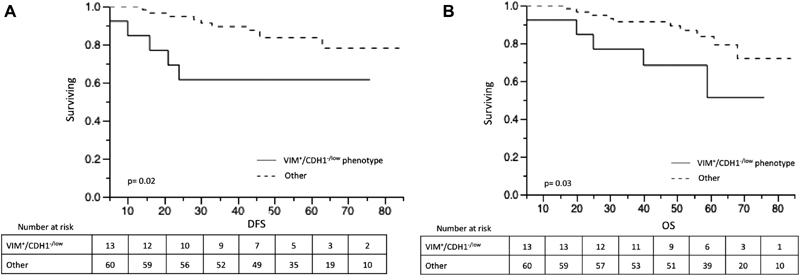
Disease-free (**A**) and overall survival (**B**) in 73 patients with invasive ductal carcinoma (IDC) of the breast according to the vimentin (VIM) and E-cadherin (CDH1) expression

We have analyzed the disease-free and overall survival according to tumor subtypes, CK5 expression, and VIM^+^/CDH1^-/low^ phenotype. We did not find a significant difference in disease-free and overall survival between luminal A, luminal B, HER2-enriched, and TNBC tumors, and there was no association between CK5 expression and survival (data not shown). In patients with VIM^+^/CDH1^-/low^ phenotype and non-VIM^+^/CDH1^-/low^ phenotype tumors, the 5-year disease-free survival rates were of 61.5% and 83.7%, and the 5-year overall survival rates were 51.2% and 83.5%, respectively. Fig. 2 shows the Kaplan-Meier curves for disease-free and overall survival according to the VIM^+^/CDH1^-/low^ phenotype, which were statically significant (*p* = 0.02 and *p* = 0.03 [log-rank test], respectively).

## Discussion

Breast cancer is a biologically and clinically heterogeneous disease.^1^ ^2^ ^3^ ^5^ ^7^ ^8^ ^9^ ^10^ ^15^ ^16^ ^17^ This complexity is a key factor for treatment failure.^1^ ^10^ ^11^ ^12^ ^35^ ^36^ Immunohistochemistry has been used to analyze the expression of specific proteins in cancer cells to identify subsets of tumors with specific characteristics, enabling a personalized treatment.^7^ ^8^ ^9^ ^10^ ^18^ ^26^ We have demonstrated that there is a subset of invasive breast carcinomas with characteristics of a mesenchymal VIM^+^/CDH1^-/low^ phenotype, suggesting that the use of mesenchymal markers may be important for therapeutic decisions.

Despite the fact there is yet no specific tumor marker to identify the basal-like subset of breast tumors, the development of a targeted therapy as a clinical approach to enhance systemic therapy has received great attention for breast cancer treatment.^11^ ^12^ ^37^ ^38^ ^39^ ^40^ The acquisition of a mesenchymal phenotype by epithelial malignant cells is an important step to cancer invasion and metastasis.^39^ ^40^ ^41^ ^42^ Some novel targeted therapies that interfere specifically with the EMT program are being developed.^43^ ^44^ ^45^ ^46^ ^47^ Triple negative tumors are considered a specific subtype of breast cancer with early recurrence and poor prognosis.^9^ ^10^ ^11^ ^14^ Transcriptome analysis has demonstrated that basal-like breast carcinomas exhibit a mesenchymal molecular profile.^3^ ^7^ ^8^ ^16^ ^17^ ^19^ ^21^ Thus, the use of an anti-EMT program therapy could be investigated in the treatment of TNBCs.

The use of CK5 has been proposed to identify a subset of TNBC tumors with a basal phenotype in some reports, demonstrating that CK5 expression in TNBCs could predict between 61 and 95% of basal-like subtypes as defined by transcriptome analysis.^20^ ^48^ ^49^ However, some basal-like tumors do not express this cytokeratin. Additionally, the expression of CK5 is observed in some non-TNBCs.^48^ ^49^ ^50^ In our study, we have observed CK5 positivity in 10% of luminal A, in 5% of luminal B, and in 12.5% of HER2-enriched IDCs, and we have demonstrated that only 33% of CK5+ tumors have a mesenchymal phenotype as defined by the positive expression of VIM and the reduction or absence of CDH1 expression (VIM + /CDH1^low/-^ phenotype).

In our study, a VIM + /CDH1^low/-^ phenotype was identified in 17.8% of IDCs, and this phenotype was observed only in TNBCs. We have found some features that classify the VIM + /CDH1^low/-^ phenotype as an aggressive IDC. These tumors exhibit a high Ki67 index, are generally poorly differentiated, and have a reduced incidence of lymph node metastasis, suggesting that they display a mesenchymal type of malignant tumor behavior.

Triple negative tumors are associated with a poor prognosis. In fact, some reports have demonstrated that there is a bipolar biological behavior among TNBC patients. There is a group of TNBC patients that develop early and aggressive recurrence, while other TNBC patients present with a more favorable prognosis.^51^ ^52^ ^53^ Our results suggest that the use of mesenchymal markers may be important to identify TNBC tumors associated with a poor outcome. In fact, we have observed that patients with the VIM^+^/CDH1^-/low^ phenotype have a higher rate of recurrence and a worse prognosis than patients with the TNBC non-VIM^+^/CDH1^-/low^ phenotype. However, this is a retrospective and single institution study including a relatively small number of samples. A large prospective study is necessary to confirm our findings.

## Conclusion

In conclusion, the IHC identification of a subset of IDC with mesenchymal phenotype suggests the selection of an aggressive IDC subtype and should prompt further investigation on this field.
